# Navigating Retinal Complications and Refractive Outcomes in High Myopia: A Case Report With Multi-surgical Interventions

**DOI:** 10.7759/cureus.78850

**Published:** 2025-02-11

**Authors:** Klaudia Szala, Sebastian Sirek, Dorota Wygledowska-Promienska

**Affiliations:** 1 Students’ Scientific Society, Department of Ophthalmology, Faculty of Medical Sciences in Katowice, Medical University of Silesia, Katowice, POL; 2 Department of Ophthalmology, Faculty of Medical Sciences in Katowice, Medical University of Silesia, Katowice, POL

**Keywords:** high myopia, pars plana vitrectomy, postoperative refraction, radial keratotomy, recurrent retinal detachment

## Abstract

Refraction through the ocular system is the process of bending light rays within the eye's optical system, which includes the cornea, anterior chamber, lens, and vitreous body. These structures work together to ensure precise focusing of light on the retina, enabling clear vision. Dysfunction or pathology at any level of the optical system can significantly affect the quality of vision, leading to changes in refractive status and visual acuity disturbances. A 58-year-old female patient presented to the Ophthalmology Clinic for a follow-up evaluation of her visual system after multiple previous surgeries, including radial keratotomy, multiple pars plana vitrectomies (PPV) due to recurrent retinal detachments, cataract phacoemulsification with intraocular lens implantation, and bilateral YAG-capsulotomy. Visual acuity was 0.004 in the right eye (OD) and 0.01 in the left eye (OS), with intraocular pressure of 20 mmHg in the OD and 18 mmHg in the OS. Autorefractor measurements were OD: +1.25/-4.0 ax 110° and OS: +1.25/-4.75 ax 130°. Pachymetry showed a central corneal thickness of 532μm in the OD and 518μm in the OS. Refraction measured by the WASCA (Wavefront Aberration Supported Cornea Ablation; (Carl Zeiss Meditec, Oberkochen, Germany)) wavefront aberrometer was +5.36/-2.43 ax 129° in the OD and +1.34/-4.08 ax 110° in the OS. Biometry results were 31.02 mm for the OD and 31.87 mm for the OS. High myopia presents complications that contemporary ophthalmology is capable of managing, even in its most severe stages. Advances in modern treatment methods often enable specialists to maintain functional visual acuity, ensuring patients can achieve a meaningful level of vision despite the challenges posed by advanced myopic conditions.

## Introduction

High pathological myopia, characterized by an axial length >26.5 mm and a spherical equivalent ≤−6.00 D, is a leading cause of visual impairment globally and poses significant treatment challenges due to its progressive and degenerative nature [1]. This condition is associated with complications such as posterior staphyloma, chorioretinal atrophy, retinal detachment, and myopic maculopathy, which can result in severe and irreversible vision loss if not appropriately managed [2]. Refraction of the eye is the process of bending light rays within the eye's optical system, which includes the cornea, anterior chamber, lens, and vitreous body. These structures work together to ensure precise focusing of light on the retina, enabling clear vision. Dysfunction or pathology at any level of the optical system can significantly affect the quality of vision, leading to changes in refractive status and visual acuity disturbances [1]. Current diagnostic tools, such as optical coherence tomography (OCT), fundus fluorescein angiography (FFA), and ultrasound biomicroscopy (UBM), play a critical role in the detailed evaluation and monitoring of complications associated with high pathological myopia. These modalities enable precise imaging of the retina, choroid, and sclera, allowing for the identification of structural abnormalities, progression of degenerative changes, and detection of complications like retinal detachment or myopic maculopathy, while therapeutic options, including vascular endothelial growth factor (anti-VEGF) therapy, vitrectomy, and scleral reinforcement, offer potential avenues for stabilizing or improving visual outcomes.

Management strategies for high pathological myopia focus on preventing progression, addressing complications, and improving visual outcomes. However, managing high myopia with coexisting retinal complications remains complex, particularly in patients with a history of multiple surgical interventions. Postoperative challenges include recurrent retinal detachment, corneal decompensation, and progressive cataract formation, which complicate visual rehabilitation efforts.

This case report analyzes the long-term outcomes of a patient with high degenerative myopia who underwent multiple surgical procedures, including radial keratotomy, vitrectomy, and anti-VEGF treatment. It explores the impact of these interventions on visual function and refractive status while addressing the challenges of achieving satisfactory visual rehabilitation in such cases. By presenting a detailed analysis of the patient's postoperative outcomes, this report aims to provide insights into the management of high myopia complicated by severe retinal pathology.

## Case presentation

The 58-year-old myopic patient underwent radial keratotomy (RK) at the age of 27 to correct a bilateral refractive error of -16 diopters (D). In the case of our patient, 10 years after the procedure, during a follow-up visit, the measured refraction was -3 D in the right eye (OD) and -3.5 D in the left eye (OS). Visual acuity was assessed as 0.5 in the OD and 0.7 in the OS. The endothelial cell density measurements were as follows in the right eye (OD): 1584 cells/mm² and in the left eye (OS): 1782 cells/mm². Pachymetric measurements were 0.485 mm in the OD and 0.475 mm in the OS. Two years earlier, elevated intraocular pressures were identified and were found to correlate with significant visual field limitations. Retinal sensitivity was notably reduced in both eyes, emphasizing the need for careful assessment and management. In the right eye (OD), a relative arcuate scotoma was observed paracentrally in the superior field, accompanied by an enlarged blind spot, highlighting localized visual field defects. In the left eye (OS), a relative arcuate scotoma was present paracentrally in both the superior and inferior fields, with continuity to the blind spot, further underscoring the need for comprehensive visual field evaluation. The treatment with 0.5% betaxolol was initiated, resulting in a reduction of intraocular pressure during the follow-up visit to 10 mmHg and 11 mmHg in the OD and OS, respectively. The patient underwent routine ophthalmic check-ups to monitor the condition of her eyes. During an optical coherence tomography (OCT) examination, the presence of an epiretinal membrane (ERM) in the right eye was observed.

The ophthalmic condition of the patient began to significantly deteriorate 20 years after the RK procedure. The complete history of performed procedures and functional test results are presented in Table 1.

**Table 1:** Complete history of performed procedures and functional test results CNV: choroidal neovascularization; PPV: pars plana vitrectomy; PVR: proliferative vitreoretinopathy; YAG: yttrium aluminum garnet; ILM: internal limiting membrane; IOL: intraocular lens

Timeline	Eye	Condition/Procedure	Refraction	Visual Acuity (VA) check-up	Endothelial cell density (ECD)	Pachymetry	Intraocular pressure (IOP)	Biometry	Additional notes
Age 27	Both	Radial keratotomy (RK)	-16 D (initial)	N/A	N/A	N/A	N/A	N/A	Bilateral surgery for high myopia
10 years post-RK Age ~37	OD	Follow-up	-3 D	0.5	1584 cells/mm²	485 um	10 mmHg	N/A	Routine check-up
	OS		-3.5 D	0.7	1782 cells/mm²	475 um	11 mmHg	N/A	
Age ~47	OD	Retinal detachment (RD), lattice degeneration, macular hole: PPV, cataract surgery	-3.5 D	0.4	N/A	528 um	16 mmHg	31.4 mm	Silicone oil tamponade, ILM peeling, endolaser photocoagulation, IOL implantation
	OS	Chorioretinal atrophy, suspected CNV	-4,0 D	0.6, gradual deterioration	N/A	525 um	18 mmHg	30.7 mm	Treated with anti-VEGF injections
Age ~48	OD	Follow-up	- 0.5/-5.0 ax 122°	0.02	N/A	N/A	15 mmHg	N/A	Gradual deterioration of vision
	OS	Cataract surgery, IOL implantation, PPV for ERM removal	+2.75/-4.5 ax 136°	0.3	N/A	N/A	16 mmHg	31.29 mm	
Age ~53	OD	Follow-up	+2.0	0.02	N/A	N/A	13 mmHg	N/A	
	OS	Macular hole, PVR, vitrectomy with SF6	+5.5/-2.0 ax 130°	0.05	N/A	N/A	15 mmHg	N/A	ILM removal, central vitrectomy
Age ~56	OD	Follow-up	+1.34/-4.08 ax 110°	0.004	N/A	532 um	18 mmHg	N/A	
	OS	Second RD, PPV with silicone oil	+5.36/-2.34 ax 129°	0.006	N/A	518um	21 mmHg	N/A	Perfluorodecalin and endophotocoagulation used
2 years post-re-RD Age ~58	OD	YAG-capsulotomy	+1.25/-4.0 ax 110°	0.004	N/A	N/A	19 mmHg	N/A	Posterior lens capsular opacification treated
	OS	Follow-up	+1.25/-4.75 ax 130°	0.01	N/A	N/A	20 mmHg	N/A	
Current Age ~58	OD	Follow-up	+1.34/-4.38 ax 114°	0.004	N/A	540 um	20 mmHg	31.02 mm	Functional assessment
	OS		+0.84/-2.87 ax 119°	0.01	N/A	529 um	18 mmHg	31.87 mm	

The first episode of retinal detachment (RD) with a giant tear and a macular hole occurred in the right eye due to blunt trauma when the patient was 47 years of age. The lattice degeneration in the right eye was also detected during the examination. A pars plana vitrectomy was performed along with simultaneous cataract phacoemulsification and intraocular lens (IOL) implantation. The procedure included the removal of vitreous hemorrhage and vitreoretinal traction. An internal limiting membrane (ILM) peeling was conducted, and peripheral endolaser photocoagulation was applied to secure the giant retinal tear along the lattice degeneration (LD). A silicone oil endotamponade was performed.

**Figure 1:**
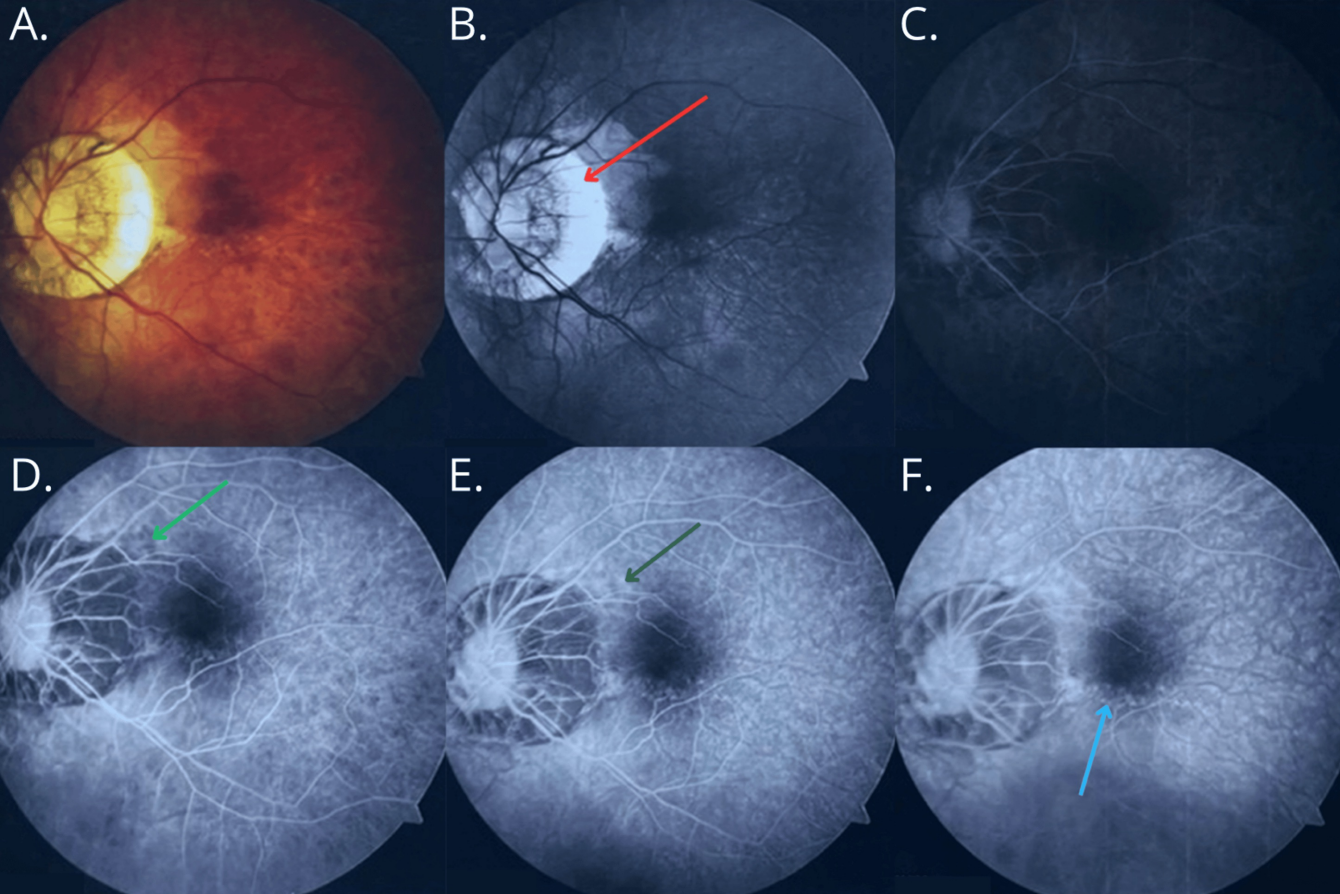
Fluorescein angiography of the fundus of the left eye Retinal circulation is normal in all phases of the angiogram. The optic disc (n.II) shows normal fluorescence and is surrounded by an extensive area of chorioretinal atrophy (red arrow). Increased choroidal background fluorescence is observed. In the posterior pole during the early phases of the examination, focal areas of patchy hypofluorescence are noted (light green arrow), which subsequently exhibit hyperfluorescence in the late phases (dark green arrow), with well-defined borders corresponding to complete atrophy of the retinal pigment epithelium. In the macula, below and nasal to the optic disc, there is a region of patchy hyperfluorescence with well-defined borders (blue arrow), possibly corresponding to a subtle neovascular membrane. A. Standard fundus photograph, B. Choroidal phase, C-D. Arterial phase, E. Arteriovenous phase, F. Late phase (elimination phase)

Fluorescein angiography of the fundus revealed an extensive area of chorioretinal atrophy and raised suspicion of a neovascular membrane (CNV) in OS (Figure 1). Due to retinal pigment epithelium (RPE) dysfunction, the patient was treated with intravitreal anti-VEGF injections.

Later, the patient required cataract phacoemulsification with intraocular lens (IOL) implantation within the capsular bag in the left eye. A pars plana vitrectomy (PPV) procedure was performed with the removal of the epiretinal membrane (ERM). Progressive deterioration of vision was observed over the years.

**Figure 2:**
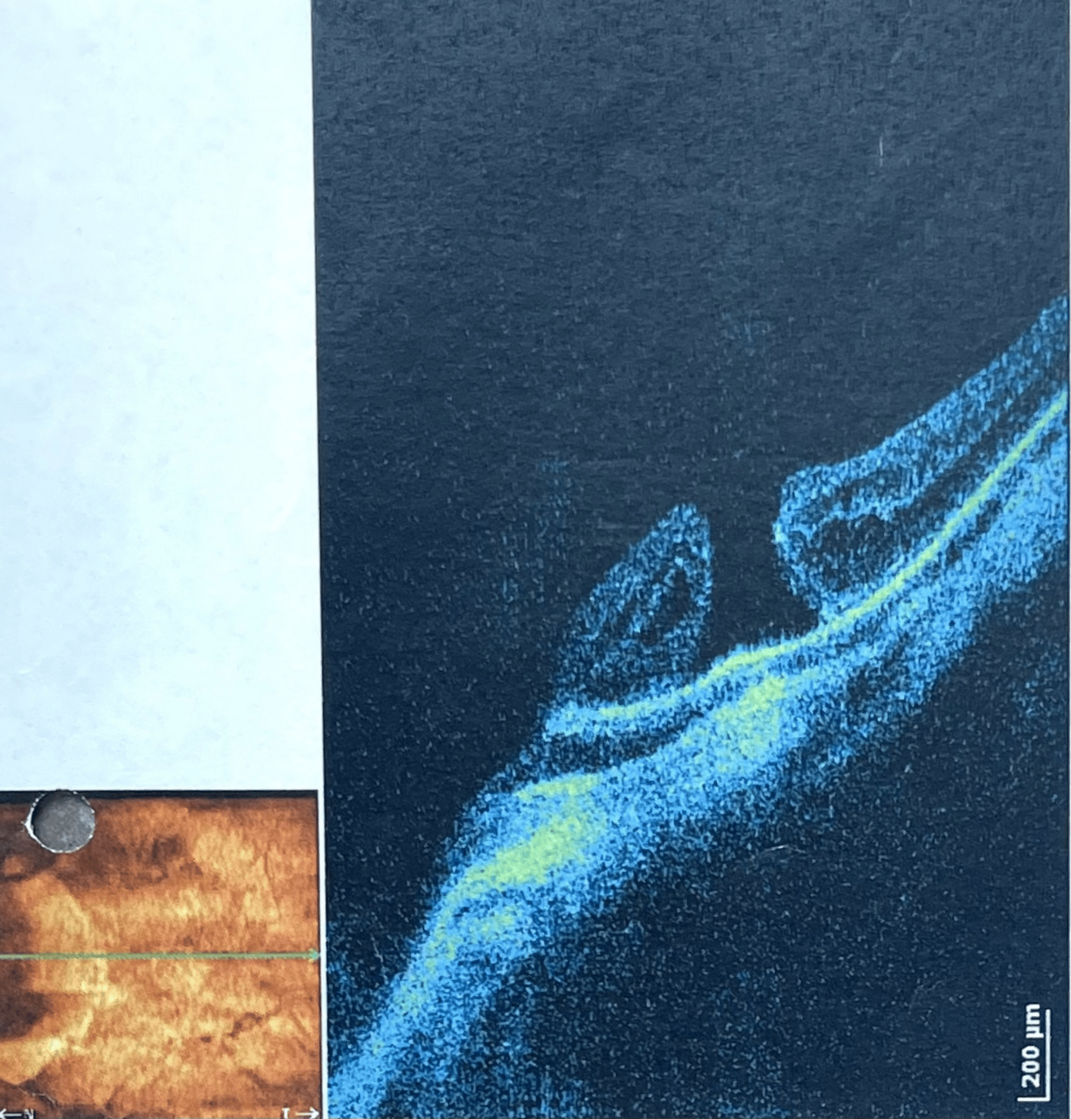
Full-thickness macular hole in the left eye (OS)

Subsequent, due to a macular hole (Figure 2) and proliferative vitreoretinopathy (PVR) in the left eye, a central vitrectomy was performed with the use of sulfur hexafluoride (SF6) gas endotamponade. During the surgery, the ILM was removed.

**Figure 3:**
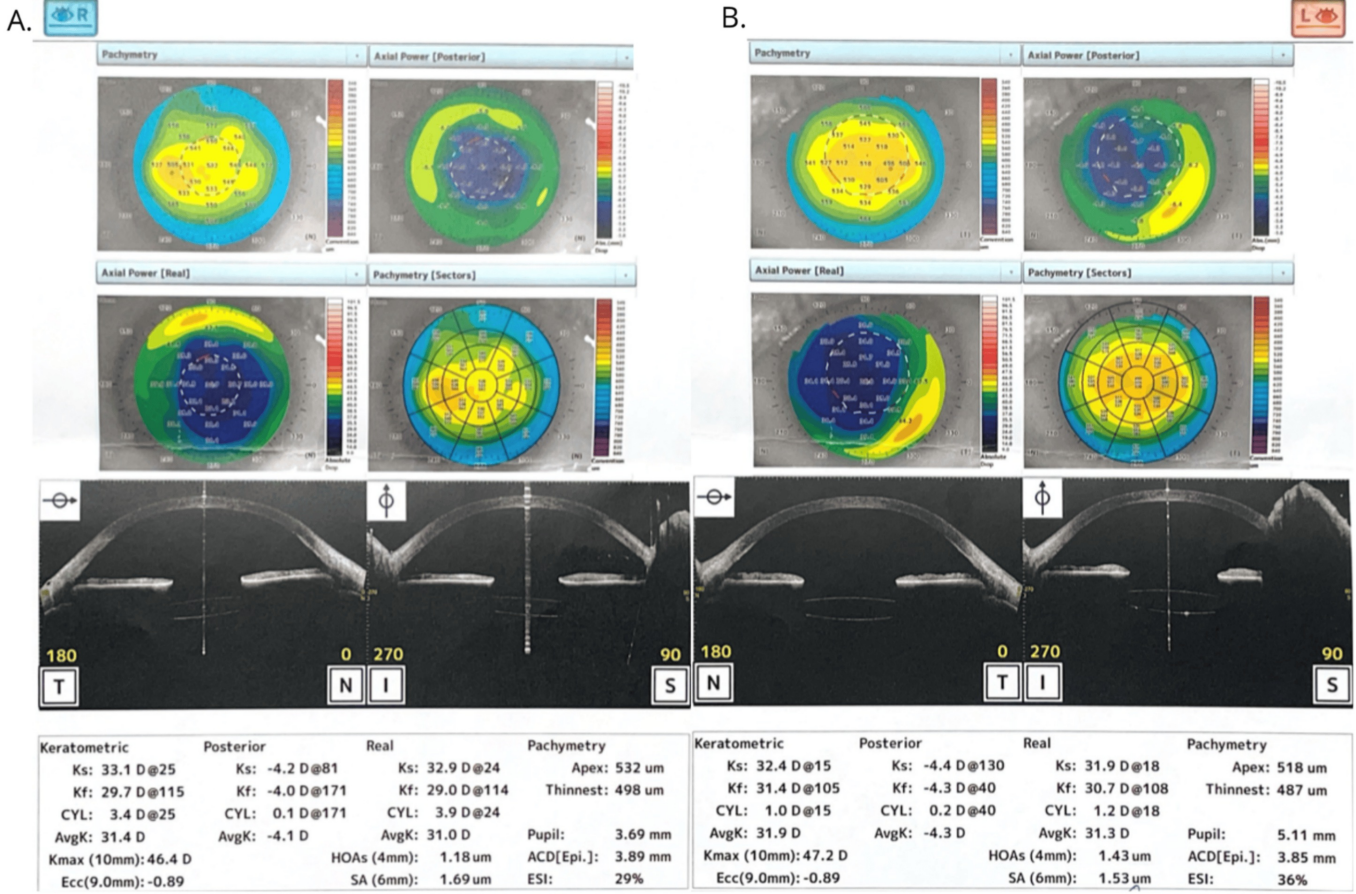
OCT examination of an anterior segment and pachymetric measurements A. Right eye OCT examination. Pachymetry: 532um. B. Left eye OCT examination. Pachymetry: 518um OCT: optical coherence tomography

**Figure 4:**
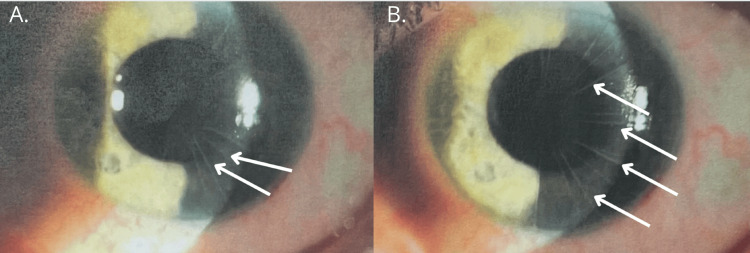
Photos of the left eye made during a slit-lamp examination A-B. Visible scars from radial keratotomy (white arrows)

After years, the patient experienced another episode of retinal detachment in the left eye. Pars plana vitrectomy with the use of perfluorodecalin, silicone oil injection, and retinal endophotocoagulation was conducted. An anterior segment OCT examination was performed, and pachymetric measurements were assessed (Figure 3). Scars from radial keratotomy were revealed during the slit-lamp examination (Figure 4).

After the last re-RD, a posterior capsulotomy with YAG laser, used as a therapy for posterior lens capsular opacification, was performed in the OD at the outpatient follow-up visit.

**Figure 5:**
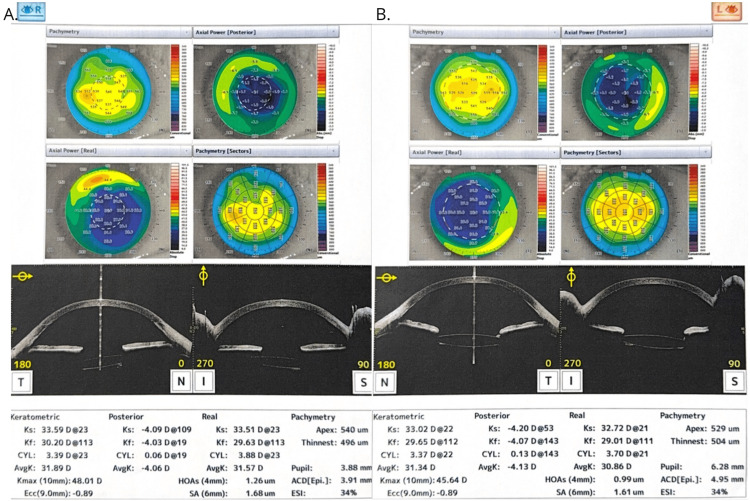
OCT examination of an anterior segment and pachymetric measurements. A. Right eye OCT examination. Pachymetry: 540um. B. Left eye OCT examination. Pachymetry: 529um OCT: optical coherence tomography

Currently, the functional results achieved in the patient are as follows: the visual acuity is 0.004 in the OD and 0.01 in the OS, while the intraocular pressure is stable (20 mmHg in the OD and 18 mmHg in the OS) due to continuous anti-glaucoma treatment (actually a combination of a carbonic anhydrase inhibitor and a β1-blocker). The patient's refraction measurements are +1.34/-4.38 ax 114° and +0.84/-2.87 ax 119° in OD and OS, respectively. The most actual OCT examination with pachymetric measurements is shown in Figure 5.

## Discussion

Myopia, particularly high myopia, is now widely recognized as a significant public health issue. Myopia is defined as a condition in which the spherical equivalent refractive error (SER) of the eye is ≤−0.50 D while high myopia is characterized by a spherical equivalent of ≤−5.00 D or ≤−6.00 D, measured when the eye’s accommodation is at rest [1,2]. High myopia is found to cause a range of specific complications such as cataract development, retinal detachment due to peripheral retinal tears, myopic foveoschisis, macular holes with or without associated retinal detachment, peripapillary deformation, dome-shaped macula, thinning of the choroid and sclera, myopic choroidal neovascularization, and glaucoma [3]. In adults, myopia leads to substantial decreases in productivity and has a detrimental effect on quality of life (QOL). Uncorrected myopia, high myopia, and the complications associated with high myopia all negatively impact QOL [2]. In the mid-1980s and early 1990s, radial keratotomy (RK) was the most widely performed refractive surgery. Despite its promising refractive outcomes, RK led to complications that were often difficult to manage or even untreatable, along with serious side effects. The most common persistent complications are overcorrection and under-correction (more frequently) [4]. Refractive side effects may include anisometropia (unequal vision between the eyes), worsening astigmatism, and symptomatic presbyopia (difficulty focusing on near objects in middle age). Other frequent but typically less severe effects include extended periods of unstable vision and mild glare. Rare but potentially serious complications involve ocular infections and corneal rupture at the keratotomy scar sites due to trauma [4].

Even the most successful RK procedures permanently altered the natural optical behavior of the cornea and its long-term biomechanical stability, making it more vulnerable to blunt trauma due to the inherent and persistent weakness of the surgical wounds [5]. Today, we know that RK is a method that has been largely replaced by more precise and safer techniques, such as LASIK (laser-assisted in situ keratomileusis) and SMILE (small incision lenticule extraction), which offer better long-term stability and fewer complications.

Rhegmatogenous retinal detachment (RD), the most prevalent form of RD, occurs when liquefied vitreous enters the subretinal space through a retinal tear, leading to the separation of the neurosensory retina from the retinal pigment epithelium [6].

The literature describes cases of retinal detachment following refractive surgery procedures, including radial keratotomy but also LASIK or LASEK [7,8].

In myopic patients with a history of refractive surgery, myopia itself may play a more significant role in the development of rhegmatogenous retinal detachment (RD) than the surgical procedure [8]. Kang HM et al. compared the characteristics of rhegmatogenous retinal detachment (RD) among patients with previous LASIK, LASEK, and myopic patients without refractive surgery. The results confirmed that myopia is a well-established risk factor for rhegmatogenous RD and may play a greater role in its development in myopic patients after refractive surgery than the surgical procedure itself [8].

Another issue in the analysis of the case we presented is the calculation of implanted lenses during the cataract surgery. It is extremely important and becomes more challenging as the patient has more comorbidities. The precision of IOL power calculation can be enhanced by aiming for myopia as the desired refractive outcome after cataract surgery and by utilizing a flatter calculated K in the IOL assessment [9]. Douglas D et al. found the Binkhorst and the Holladay intraocular lens calculation formulas to be more accurate than the SRK II for patients undergoing cataract extraction with posterior chamber lens implantation several years after radial keratotomy [7].

It is believed that an increase in globe size leads to thinning and stretching of the sclera, which contributes to changes in the fundus. However, the precise mechanism behind these fundus changes remains unclear. Myopic alterations in the retina, choroid, and optic nerve head (ONH) are frequently observed in cases of high myopia [10]. Axial length (AL) elongation and the subsequent development of posterior staphyloma are central events contributing to the structural changes associated with high myopia [11]. Additionally, peripapillary and subfoveal choroidal thinning, scleral thinning, and irregular or deformed eye shapes have been strongly linked to various pathological myopic lesions, particularly posterior staphyloma and chorioretinal atrophy [12]. In the context of the described case, the occurrence of chorioretinal atrophy is highly likely to correlate with globe elongation (axial length >30 mm) [13].

The pachymetry measurements, as well as the differences observed in the case we describe, may stem from variations in the equipment used for the measurements. The initial available pachymetry result was obtained using the ultrasonic pachymetry method, while the most recent measurements were performed using the Pentacam device. The time difference between the first and last available measurement is approximately 20 years, during which the patient underwent multiple surgical procedures, including PPV, with the use of various types of endotamponades. The ultrasound pachymetry (USP) technique remains one of the most commonly employed methods for obtaining quick, accurate, and consistent measurements of corneal thickness at an affordable cost, despite the fact that several newer technologies have demonstrated superior repeatability and reliability for this purpose, including Pentacam, which was used by us [14]. The limitation in this area is the availability of the patient's own documentation and the inability to verify it due to treatment in multiple ophthalmology centers.

In the described case, PPV was performed simultaneously with peripheral endophotocoagulation to secure the detachment site and prevent another episode, which proved to be effective (no recurrence of detachment occurred, affecting both the right eye and the left eye, where endophotocoagulation was decided upon during the second detachment). Studies indicate that many surgeons opt to perform cataract extraction during PPV, even when the cataract is not clinically significant [15,16]. This is because cataract development in phakic eyes following PPV is a common outcome, with reported rates ranging from 17% to 80% [17].

Hamoudi et al. explored the impact of different surgical sequences on the count and morphology of corneal endothelial cells (EC) and the biomechanical properties of the cornea. Their study compared combined phaco vitrectomy and two-step sequential surgery (either cataract surgery first, followed by vitrectomy, or vice versa). The findings revealed that all groups experienced significant EC loss after surgical interventions. However, as expected, corneal endothelial cell density (CED) significantly decreased only after cataract surgery, not vitrectomy. The EC loss was slightly lower in the group undergoing cataract surgery and subsequent PPV (the CAT group), but this difference was not statistically significant compared to other strategies. One possible explanation is that the cataracts in these cases were often only in an early stage at the time of surgery [18]. This observation is also relevant in combined surgeries (phacovitrectomy), which may be performed even when the lens remains clear, as cataract formation and progression are frequent outcomes following vitrectomy [19]. With regard to the patient we describe, a limitation is the lack of data describing CED; however, remaining under the care of our center, we are considering performing such a study for comparative purposes.

## Conclusions

The case of this 58-year-old patient with high degenerative myopia and a history of multiple surgical procedures, including radial keratotomy, pars plana vitrectomy, and cataract surgery, highlights the complex relationship between refractive outcomes and postoperative visual acuity. Despite the challenges posed by these extensive ocular interventions, including retinal detachments and complications like proliferative vitreoretinopathy, the possibility of preserving functional visual acuity remains. The patient's refractive status was significantly influenced by prior surgeries, particularly radial keratotomy and the use of endotamponades such as silicone oil. These interventions caused alterations in the eye's optical properties, which were reflected in the postoperative refraction. However, with ongoing management, including cataract surgery and precise IOL power calculation, some functional visual acuity was maintained.

The limitations of this case management stem from the cumulative impact of multiple surgical interventions, which significantly altered the ocular anatomy and optical properties, complicating the predictability of refractive outcomes. The patient's prior radial keratotomy introduced corneal irregularities and unpredictable refractive changes, making it challenging to achieve optimal postoperative visual acuity. Additionally, the use of endotamponades such as silicone oil contributed to refractive changes and posed long-term risks. These factors resulted in refractive instability, further complicating the ability to predict and achieve the desired refractive outcome. Despite efforts to restore visual function, the structural damage caused by complications such as retinal detachments and proliferative vitreoretinopathy limited the achievable postoperative visual acuity. This case underscores the importance of careful postoperative management, personalized refractive strategies, and long-term follow-up to optimize the functional outcomes for patients with high myopia and multiple ocular surgeries.
